# Acceptability of donor breast milk banking, its use for feeding infants, and associated factors among mothers in eastern Ethiopia

**DOI:** 10.1186/s13006-018-0163-z

**Published:** 2018-06-26

**Authors:** Tilayie Feto Gelano, Yadeta Dessie Bacha, Nega Assefa, Aboma Motumma, Aklilu Abrham Roba, Yohanes Ayele, Fikirte Tsige

**Affiliations:** 1 Department of Pediatrics and Neonatal Nursing, School of Nursing and Midwifery, College of Health and Medical Sciences, Haramaya University, P.O. Box, 235, Harar, Ethiopia; 2 School of Public Health, College of Health and Medical Sciences, Haramaya University, Harar, Ethiopia; 3 School of Nursing and Midwifery, College of Health and Medical Sciences, Haramaya University, Harar, Ethiopia; 4 School of Pharmacy, Department of Clinical Pharmacy, College of Health and Medical Sciences, Haramaya University, Harar, Ethiopia; 5 School of Medicine, Department of Pediatrics, College of Health and Medical Sciences, Haramaya University, Harar, Ethiopia

**Keywords:** Human milk banking, Donated human breast milk, Eastern Ethiopia

## Abstract

**Background:**

The first priority for infant feeding is to encourage the use of infant’s mother’s breast milk, but when this is not possible, donated breast milk is the second best option. In developing countries, very few studies have been conducted on the acceptance of donor breast milk. Hence, this study was planned to discover the acceptability of donor breast milk banking, its use for feeding infants, and associated factors among mothers in eastern Ethiopia.

**Methods:**

A mixed method study was conducted in eastern Ethiopia from December 2015 to February 2016. Data were collected through a pre-tested structured interview based questionnaire. A total of 1085 mothers participated in the survey and six focus group discussions were held with 33 mothers. Descriptive statistics have been used to report results from the survey and qualitative data were analyzed using the thematic data analysis approach.

**Results:**

The study revealed that 119 (11%) of participants were willing to donate breast milk for banking and 165 (15.2%) of mothers were willing to use for feeding infants. The acceptance of donor milk banking was 5.8 times more likely among the mothers who had heard about donor milk banking previously (Adjusted Odds Ratio [AOR] 5.8; 95% Confidence Interval [CI] 3.1, 10.72), 4.2 times more likely among the mothers who heard about wet-nurses (AOR 4.2; 95% CI 2.5, 6.99) and 2 times more likely among mothers who had visited a neonatal intensive care unit (AOR 2; 95% CI 1.1, 3.73).

**Conclusions:**

Generally, this study showed that the acceptance of breast milk donation for banking and its use for feeding infants was very low, due to lack of information and misconceptions about the safety of breast milk. Therefore, before initiation of any donor milk banking program awareness should be created about donor breast milk and its safety.

## Background

Human milk banking is the process by which breast milk is collected, screened and pasteurized for the use of hospitals or mothers who cannot breastfeed [1]. The first human milk bank was found in 1909 in Vienna, Austria, and shortly afterwards in Boston, United States of America. Currently, many milk banks have been opened all over the world to minimize infant feeding problems [2].

Human milk is the best source of nutrition for all newborn babies. More specifically, a mother’s breast milk is the first choice of nutrition for those who are preterm, have low birth weight, are unwell [3] and for those vulnerable infants in the Neonatal Intensive Care Unit (NICU) [2, 4–6].

Pasteurized donor breast milk (PDBM) is not the same as fresh breast milk as it loses certain bioactive and immunological properties [7–9]. The ingredients of human breast milk include immunoglobulins and other active constituents that can reduce infection, necrotizing enterocolitis, cardiovascular risk and metabolic diseases [3, 10, 11]. If a mother’s own breast milk is not available, the second choice should be donor breast milk [4–6, 12]. Many studies have shown that donor breast milk has short and long term benefits as compared to preterm formula [4–6]. This has also been recommended by the World Health Organization (WHO) [11].

Worldwide, 4 million babies die each year in their first four weeks of life. This represents more than 10,000 deaths per day. Most neonatal deaths occur at the very beginning of life; three-quarters of them occurring within one week of birth [13, 14]. The three major causes of neonatal mortality are severe neonatal sepsis (36%), prematurity (28%), and birth asphyxia (23%) [13, 14].

In Ethiopia, the Neonatal Mortality Rate (NMR) has always been very high with an estimated 63% of infant deaths occurring during the first month of life. According to the Ethiopian Demographic and Health Survey (EDHS) of 2011 report, the Neonatal Mortality Rate accounted for 42% of Under-Five Mortality (U5MR) [13]. In spite of many efforts made for the improvement of maternal and child health care services by the government and other stakeholders, the reduction of NMR has remained insignificant. For the years 1991–1995 NMR was reduced from 46 per 1000 live birth to only 42 for 1996–2000, to 39 for 2001–2005, and to 37 for 2006–2011 [14–16]. In Ethiopia, a study conducted in 2007 and 2013 also indicated that prematurity (26.4%), pneumonia (22.6%), neonatal tetanus (9.4%) and sepsis (7.5%) were the leading causes of neonatal mortality [14, 16].

Breastfeeding promotion and the collection of donor breast milk are linked. By offering correct information about breastfeeding, women have an increased chance of successfully breastfeeding their infant [15]. Having a human milk bank in a health facility increases awareness about breastfeeding among families and the community [17]. It is also known that successful breast feeding significantly reduces neonatal mortality and morbidity worldwide [18, 19]. In addition, the availability of donor breast milk is very significant for infants whose mother cannot breastfeed because of medical problems such as maternal open pulmonary tuberculosis, cancer chemotherapy, HIV and other viral infections [1, 3]. To fulfil this need, establishing donor breast milk banking is crucial.

In Africa, very few studies have been conducted on the acceptance of breast milk donation for banking and use of donor breast milk for feeding infants [5, 20, 21]. In Ethiopia, no study has been conducted on this topic. Hence, these conditions have prompted us to conduct the study on the acceptance of breast milk banks for feeding infants among mothers who were attending selected public hospitals in eastern Ethiopia.

For the purposes of the study, acceptance of breast milk banks was measured by the willingness of mothers to donate breast milk or to use the pasteurized donated breast milk for feeding their infants.

## Methods

A descriptive cross-sectional, mixed method study was conducted. Data were collected through surveys and Focus Group Discussions (FGDs). The study was conducted among pregnant and breastfeeding mothers, who were receiving care during the data collection, in Hiwot Fana Specialized University Hospital, Jugola Hospital, Dil-Chora Hospital, and Karamara Hospital, all of which are public hospitals in eastern Ethiopia. The study took place between December 2015 and February 2016.

Hiwot Fana Specialized University Hospital, established in 1941provides service for more than 154,196 patients a year and Jugola Hospital, which provides service for more than 90,000 patients a year, are found in Harar. Harar is the capital city of the Harari Regional State and is 517 km away from Addis Ababa. Hiwot Fana Hospital was handed over to Haramaya University in 2010 and since then it has been named as Hiwot Fana Specialized University Hospital [22, 23].

Karamara Hospital is found in Jigjiga City, a capital city of Somali Region and is located 635 km from Addis Ababa, the capital city of Ethiopia. It provides service to more than 90,500 patients per year [24]. Dil-Chora Hospital, which provides service to more than 91, 250 patients per year is located in Dire-Dawa Administrative City, which is 527 km from Addis Ababa [25].

### Source and study population

All the breastfeeding and pregnant mothers who were receiving care at the selected public hospitals in eastern Ethiopia during the study period.

#### Inclusion and exclusion criteria

##### Inclusion criteria

All breastfeeding and pregnant mothers who were referred from other health facilities to selected hospitals during the study period were included in this study.

##### Exclusion criteria

Critically ill mothers who could not answer survey questions were excluded from the study.

#### Sample size determination

##### Sample size for survey

One thousand and eighty five breastfeeding and pregnant mothers were involved in the study. The sample size was calculated using a single population proportion formula with 95% confidence level, and 3 % margin of error was used to get the largest sample possible for the survey. The proportion of 59.1% from a study conducted in Nigeria on mother’s attitude towards donated breast milk was used [26] and a 5 % non-response rate was estimated.

##### Sample size for qualitative data

A total of six Focus Group Discussions (FGDs) that involved 33 study participants were conducted, and each group consisted of five to seven members.

##### Sampling procedure

**Fig. 1:**
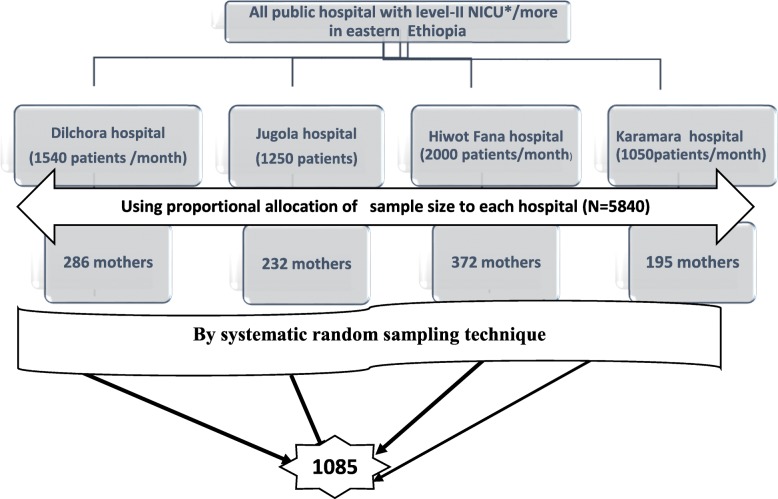
Schematic representation of sampling technique

Hiwot Fana Specialized University Hospital, Jugola, Karamara, and Dil-chora Hospitals are the only hospitals that have level two (level-II) or more Neonatal Intensive care Unit (NICU) service in eastern Ethiopia during the study period, and all of them were included in this study. Level-II NICU is also called specialty care and provides services for premature, low birth weight and sick newborn infants [27]. The sample size was allocated to these institutions based on the average number of patients they treat monthly. Finally, the study subjects were selected by using systematic random sampling method, by considering calculated K-value of five (Fig. 1).

##### Data collection methods and tools

Data on the socio-demographic characteristics of the participants, maternal health care service use, acceptance of donor breast milk banking, and its use for feeding infants were collected via a pretested, structured interview based questionnaire.

For qualitative data, the topics or themes which were used to guide the FGDs were *awareness of mothers towards human breast milk banking*, *importance of human breast milk banking and acceptability of human breast milk donation for banking and the use of donated breast milk for feeding infants.* The length of time for each group discussion was determined by point of saturation and the maximum length was two hours. Each group discussion began with the explanation of the purpose of the study and obtaining informed voluntary consent from the participants. The discussion was structured as beginning (getting people talking, relating experiences and ideas), middle (helping people to focus by asking them more specific questions on the topics) and ending or completing group task. An MSc nurse who was trained for this purpose directed the discussions while the principal investigator was tape recording and taking notes.

##### Ensuring data quality

After we had reviewed similar studies, the structured interview-based questionnaire was developed. The tool was first prepared in English and then translated into the local languages (Afan Oromo, Amharic and Somali) for an interview purpose, and finally it was translated back into English for the data analysis. The questionnaire was pretested on 5 % of the total sample size at a different area from the actual data collection site. The data were collected by eight BSC nurses who were given a two-day intensive training course on the content of data collection tools, objectives and how to interview the study subjects. The collected data were checked for completeness each day and any possible errors were returned to the data collectors for correction.

For qualitative data collection, the research team reviewed the guiding questions for face validity. They were pilot-tested with two individuals and modified based on these pilot discussions to create the final version. The moderator was trained on the topics or themes which guided the discussions. All discussions were conducted in the local languages (Oromiffa, Amharic, and Somali). During discussion, the chairs were arranged in a U-shape, with every participant visible to each other and to the moderator behind who was tape recording and the principal investigator who was note taking.

##### Data analysis and processing

Data obtained from the survey were checked for completeness, coded and entered into EPI-Info7 and imported into SPSS Version 20 for analysis. They were cleaned and prepared for tabulation. Then, frequency and other descriptive statistics (graphs, charts, proportion, mean and standard deviation) were conducted. The acceptance of donor breast milk among the participants was computed from two variables: Mothers’ willingness to donate breast milk for banking and /or mothers’ willingness to use donated breast milk for infant feeding. A mother was considered as accepting of donor milk banking if she was willing to donate breast milk for banking and /or she was willing to feed donor breast milk to her infant. Any mother who selected both (willingness to donate breast milk for banking and willingness to use donor milk for feeding infant) was counted once. Bivariate analysis was conducted to examine the association between dependent and independent variables; Crude Odds Ratios (COR) and their 95% CI, were calculated. Then, all the variables which have *p*-value less than 0.2 in the bivariate analysis were included in the multivariate binary logistic regression model to identify explanatory variables that were associated with the acceptance of donor milk banking. Adjusted Odds Ratios (AOR) were computed with 95% Confidence Intervals (CI) and p-value of less than 0.05 was considered to identify significant association. The data obtained from the FDGs were first transcribed and then translated into English language by replaying the record. Finally, they were analyzed using thematic approach by organizing the same concept raised at the discussions.

#### Study variables

##### Dependent variables

Acceptance of breast milk donation for banking.

Acceptance of using donated milk for feeding infants.

##### Independent variables

Socio-demographic characteristics of the mothers.

Health care service use of the mothers.

Awareness of the mothers about human breast milk banking.

#### Measures

##### Human breast milk banking /HBMB

Refers to a service which collects, screens, processes, and dispenses donor breast milk to hospitals or recipients.

##### Acceptance of breast milk donation

Refers to a mother’s willingness to donate breast milk for banking.

##### Acceptance of use of donor milk for feeding infant

Refers to a mother’s willingness to use pasteurized donated breast milk for feeding her infant.

##### Acceptance of donor milk banking

A mother was considered to accept donor breast milk banking if she had willingness to donate breast milk for banking and/or she had willingness to use donor breast milk for feeding her infant.

##### Awareness of mothers about donor milk banking

A mother was considered to be aware about donor breast milk banking if she had ever heard about donor milk banking.

##### Human milk donation

Refers to the act of the lactating mother to give breast milk for human milk banking.

##### Wet-nurse

Refers to a mother who breast feeds for another’s baby.

## Results

### Socio-demographic characteristics

Of the 1085 mothers who were surveyed at the four selected hospitals, the mean age was 27.7 years ± SD 4.7, 68.8% lived in urban areas, 90.7% were between 18 to 34 years old, 95.6% were married and 75.2% were literate (Table 1).

**Table 1:** Socio-demographic characteristics of pregnant and breastfeeding mothers attending selected public hospitals in Eastern Ethiopia, 2016 (*N* = 1085)

Variables	Category	Frequency	Percent (%)
Residence	Urban	746	68.8
	Rural	339	31.2
Ethnicity	Oromo	428	39.4
	Amhara	241	22.2
	Somale	218	20.1
	Harari	105	9.7
	Tigray	35	3.2
	Other^a^	58	5.3
Age in years	18–34 years	984	90.7
	35–48 years	101	9.3
Religion	Orthodox	302	27.8
	Muslim	711	65.5
	Protestant	57	5.3
	Catholic	15	1.4
Educational status	Illiterate	269	24.8
	Literate	816	75.2
Occupation participants	House wife	646	59.5
	Trading/merchant	135	12.4
	Government employed	195	18.0
	Private employed	109	10.1
Marital status	Married	1037	95.6
	Unmarried	23	2.1
	Separated^b^	25	2.3
Husband educational status	Illiterate	156	14.7
	Literate	904	85.3
Husband occupation	Farmer	280	26.6
	Government employed	385	36.6
	Merchant	212	20.1
	Private employed	122	11.6
	Daily laborer	54	5.1
Monthly income	+ < 1000 Birr	166	15.3
	1001–2500 Birr	276	25.4
	> + 2501 Birr	643	59.3

^*a*^ *Gurage and Sidama*

^*b*^ *Divorced and widowed*

### Maternal characteristics and health care services use

The mothers were interviewed about their health care service use for their last pregnancy and maternal characteristics. More than half (54.7%) of the mothers had visited a Maternal and Child Health Care unit (MCH). With regards to number of pregnancies and live births, 71.7% of the mothers had 1–3 gravida and 76.2% of them had 1–3 parity. Looking at utilization of maternal health care services, 83.1% of the mothers had had at least one antenatal visit and 84.2% of them had given birth at a health facility. Among the mothers who visited an Antenatal care unit (ANC), about 62.9% of them were counseled on breastfeeding. Of the total participants who received postnatal care (PNC) after the last pregnancy, the majority, 92.9% of them were counseled on breastfeeding and more than three quarters (76.7%) of the mothers were currently breastfeeding (Table 2).

**Table 2:** Health care services use and characteristics of study participants at selected public hospitals in eastern Ethiopia, 2016 (*N* = 1085)

Variables	Category	Frequency	Percent (%)
Visited units in the hospitals	MCH	594	54.7
	Obstetrics	164	15.1
	Pediatrics	165	15.3
	NICU	162	14.9
Number of pregnancy/gravida	1–3	778	71.7
	> + 4	307	28.3
Number of life birth/parity	1–3	827	76.2
	> + 4	258	23.8
Antenatal visits during last pregnancy	Yes	902	83.1
	No	183	16.9
Number of antenatal visits	1–3	486	53.9
	> + 4	416	46.1
Counseling on BF at antenatal visits	Yes	567	62.9
	No	335	37.1
Birth place of last child	Home	172	15.9
	Health center	235	21.7
	Hospital	678	62.5
Postnatal visits after the last pregnancy	Yes	346	31.9
	No	739	68.1
Number of postnatal visits	1–2	222	64.2
	> + 3	124	35.8
Counseled on BF at postnatal visits	Yes	320	92.5
	No	26	7.5
Currently BF	Yes	253	76.6
	No	832	23.3
Ever visited under-five OPD	Yes	881	81.2
	No	204	18.8
Counseled on BF at under-five OPD	Yes	579	65.7
	No	302	34.3
Ever experienced difficulty of BF	Yes	43	4
	No	1042	96
Condition that limited mothers from BF	Mothers’ illness	20	46.5
	Lack of breast milk	23	53.5
Alterative used for difficulty of BF	Infant formula was used	41	53.9
	cow milk was used	18	23.7
	Soft food made of cereals	17	22.4
Ever experienced BF to others’ baby	Yes	28	2.6
	No	1057	97.4

*MCH maternal & child health care unit, NICU Neonatal intensive care unit, OPD Outpatient department, BF Breast Feeding*

### Awareness of mothers about donor milk banking

Concerning awareness of mothers about human breast milk banking (HBMB), 22.3% of mothers had heard about wet nurses but only 10% of mothers had ever heard about HBMB and 5.6% of mothers stated that collecting and storing human breast milk is useful (Table 3).

**Table 3:** Awareness on donor milk banking among mothers attending four selected public hospitals in eastern Ethiopia, 2016 (*N* = 1085)

Variables	Category	Frequency	Percent (%)
Ever heard about wet nurse	Yes	242	22.3
	No	843	77.7
Ever heard about HBMB	Yes	108	10
	No	977	90
Ever experienced breastfeeding others’ infants	Yes	28	2.6
	No	1055	97.4
Collecting and storing breast milk is useful	Yes	61	5.6
	No	1024	94.4

*HBMB human breast milk banking*

Most of the participants in the FGDs stated that they did not know about human breast milk banking or donor breast milk banking. A 20-year-old breastfeeding mother stated that, “*I do not understand about human breast milk banking. In the past I have heard information about wet nurses but this is only possible if we have a blood relation with the baby’s family. For example, my sister can feed my baby if I cannot feed my baby otherwise it is not possible.”*

A 21-year-old mother said that, *“I have heard about donated human milk for infants feeding from other countries, such kinds of practices are especially present in orphanages but in our country there are no such kinds of practice.”*

### Acceptance of donating human milk for banking and its use for feeding infants

**Fig. 2:**
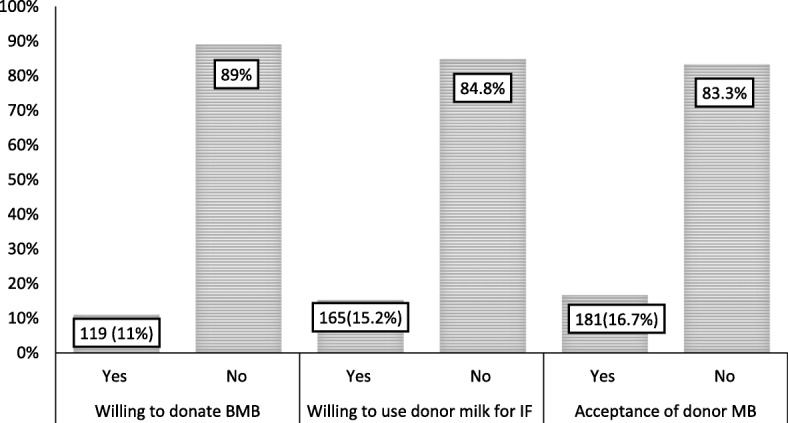
Acceptance of breast milk donation and its use for infants feeding among mothers at selected public hospitals in eastern Ethiopian, 2016 (N = 1085)

With regards to accepting breast milk donation or banking and willingness to use donated milk for feeding infants, only 119 (11%) of the participants were willing to donate breast milk for banking and 165 (15.2%) of mothers were willing to feed donated breast milk to their infant. About 181 (16.7%) of the participants accepted donor human breast milk banking (Fig. 2).

### Reasons why women would be willing to donate breast milk for banking or to utilize it for feeding infants

**Fig. 3:**
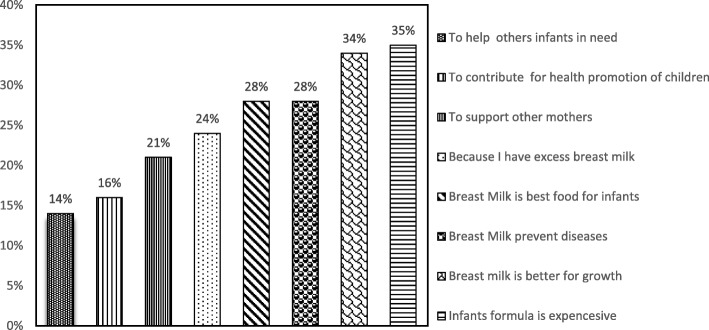
Reported reasons to accept donation of human breast milk for banking and its use for infants feeding among mothers attending selected public hospitals in eastern Ethiopia, 2016 (*N* = 1085)

With regard to reasons for being willing to donate breast milk for banking and its use for infant feeding**,** about 35% of mothers said that formula milk is expensive, 21% of them reasoned that human breast milk prevents diseases, and 28.3% of the participants stated that they had excessive breast milk (Fig. 3).

The finding from FGDS also showed that there were supporting ideas to donate breast milk and use it for infant feeding. According to the participants who were willing to donate breast milk for banking; it was stated that donated milk is good for babies who cannot get their own mothers’ breast milk.

The participants of FGDs stated that*, “If breast milk is left from my baby or if I have excess breast milk, I will give it for another baby who cannot get his mother’s breast milk. I do this because it is good to support another’s baby. It is good idea to donate breast milk for other babies if we have enough milk. For example, a mother next to my bed in this hospital (Hiwot Fana Specialized University Teaching Hospital) has no breast milk to feed for her baby because she is sick. For this kind of baby, I am happy to donate.” (*27-year-old mother). On the same point a 23-year-old mother stated that, “*Especially if the health of donor mother is checked, donating breast milk will be a solution for those mothers who cannot feed their baby.”*

### Reasons for being unwilling to donate breast milk for banking and its use for infants feeding

Reasons given in interviews for being unwilling to donate breast milk for banking or to use it for infant feeding included fear of disease transmission (68.6%), fear of unhygienic milk collection (43.9%) and religious issues (19.1%) (Table 4).

**Table 4:** Reported reasons of unwillingness to donate breast milk for banking and its use for infants feeding among mothers attending selected public hospitals in eastern Ethiopia, 2016 (N = 1085)

Variables	Category	Frequency	Percent (%)
Fear of disease transmission	Yes	746	68.8
	No	339	31.2
Fear of genetics mixing	Yes	134	12.4
	No	951	87.6
Preference of infant formula	Yes	137	12.6
	No	948	87.4
Unhygienic milk collection	Yes	476	43.9
	No	609	56.1
Spouse and family not support	Yes	91	8.4
	No	994	91.6
Fear of not having enough breast milk	Yes	556	51.2
	No	529	48.8
I do not like the idea	Yes	517	47.6
	No	568	52.4
Not accepted in our religion	Yes	207	19.1
	No	878	80.9
Not accepted in our culture	Yes	159	14.7
	No	926	85.3
Breast sagging	Yes	9	0.8
	No	1076	99.2

The finding from FGDs also showed that reasons for being unwilling to use donor breast milk for feeding infants were also due to fear of disease transmission, safety of donated breast milk, and religion. A 23-year-old mother stated that, *“I fear the transmission of different diseases from the use of donated human breast milk for infant feeding. Especially diseases such as HIV can be transmitted to baby through donated breast milk.”* On this issue a 21-year-old mother explained, *“It is not good to feed someone else’s breast milk to my baby because there are some diseases such as cancer and acne [Golfaa and Finnisa] that can be transmitted to baby through breast milk.”* Another mother stated that, *“It is not acceptable to use someone else’s breast milk for infant feeding because it is not good and the milk may expire. I do not like to donate my breast milk for other infants, because I do not think that my breast milk is good for other babies.”* (21-year-old mother).

The other concern which was mentioned by the participants was the issue of milk sharing and religion. Some of the participants from FGDs were concerned that human breast milk sharing is prohibited. A 22-year-old mother stated, *“I will not give donated breast milk to my baby; rather I prefer to use other feeds such as cow milk or formula milk than donated milk*. *It is not right to feed someone else’s breast milk to infants even if the baby cannot get his mother’s own breast milk, rather it is better to feed formula or cow milk by bottle. In the Muslim religion we do not support feeding someone else’s breast milk to our infants. Because it is prohibited.”*

### Acceptance of human milk banking and associated factors

In this study, the factors associated with the acceptance of breast milk banking were also assessed. The use of health care services, NICU visit (AOR 2; 95% CI 1.1, 3.73) and number of antenatal visits during pregnancy (AOR 0.52; 95% CI 0.32, 0.83) were associated with the acceptance of human milk banking. Acceptance of human milk banking was over 5 times more likely among mothers who had ever heard about human breast milk banking (AOR 5.8; 95% CI 3.1, 10.72) compared to those who had not heard about it, and it was 4.2 times more likely among mothers who had heard about wet-nurses (AOR 4.2; 95% CI 2.5, 6.99) compared to those who had not heard about them (Table 5).

**Table 5:** Bivariate and multivariate logistic regression to identify factors associated with acceptance of donor milk banking among mothers attending public hospitals in eastern Ethiopia, 2016 (N = 1085)

Explanatory variables	Outcome variable		COR (95% CI)	AOR (95% CI)
	Acceptance of HBMB			
	No	Yes		
Residence				
Urban	600	146	1	1
Rural	304	35	0.47 (0.32, 0.7)	0.8 (0.4, 1.6)
Educational status				
Illiterate	238	31	1	1
Literate	666	150	1.7 (1.14, 2.6)	1 (0.48, 2.2)
Participants occupation				
House wife	551	95	1	1
Trading/merchant	123	12	0.56 (0.3, 1.1)	0. 7 (0.33, 1.55)
Government employed	133	62	2.7 (1.8, 3.9)	1.7 (0.97, 2.95)
Private employed	97	12	0.72 (0.4, 1.4)	0.84 (0.35, 2)
Visited units in the hospital				
MCH	509	85	1	1
Obstetrics ward	138	26	1.1 (0.7, 1.8)	1.33 (0.66, 2.68)
Pediatrics ward	146	19	0.78 (0.46, 1.3)	0.98 (0.47, 2.1)
NICU	111	51	2.7 (1.8, 4.1)	2 (1.1, 3.73)
Antenatal visits during last pregnancy				
No	163	20	1	1
Yes	741	161	1.77 (1.08, 2.9)	1.4 (0.8,2.9)
Number of antenatal visits				
1–3 ANC visits	383	103	1	1
> + 4 ANC visits	357	59	0.62 (0.43, 0.87)	0.52 (0.32, 0.83)
Counseling about BF during antenatal visits				
No	293	42	1	1
Yes	448	119	1.85 (1.3, 2.7)	2.2 (1.2, 4.2)
Postnatal visits after last pregnancy				
No	628	111	1	1
Yes	276	70	1.4 (1, 2)	1.1 (0.64, 1.84)
Counseling of BF at under-five OPD				
No	268	34	1	1
Yes	465	114	1.9 (1.3, 2.9)	0.75 (0.38, 1.46)
Ever heard about HBMB				
No	858	119	1	1
Yes	46	62	9.7 (6.3, 14.8)	5.77 (3.1, 10.72)
Ever heard about wet nurse				
No	762	81	1	
Yes	142	100	6.6 (4.6, 9.3)	4.2 (2.5, 6.99)

*Significant at P < 0.001, HBM human breast milk, HBMB human breast milk banking, MCH* maternal and child health care unit, *NICU* Neonatal intensive care unit, *ANC* Antenatal care, *PNC* Postnatal care, *OPD* Outpatient department, *BF* Breast Feeding, *COR* Crude Odds Ratio, *AOR* Adjusted Odds Ratio

FGD data also indicated that mothers who had their newborn baby admitted to a Neonatal Intensive Care Unit supported breast milk donation and its use for infant feeding. Some mothers also stated that it is good to donate breast milk for babies who will not receive their own mothers’ breast milk.

## Discussion

The current study sought to determine the willingness of mothers to donate their breast milk for breast milk banking and their acceptance of its use for infants feeding. The survey showed that among total study participants only 119 (11%) were willing to donate breast milk for banking. This percentage is less than the study report from Nigeria, which showed that 39.9% of the study subjects accepted breast milk donation [21]. The difference might be due to the fact that in Nigeria the service has already started and this might have increased the awareness of the mothers, unlike most of the participants in our study (90%) who said they had no information about human milk banking or donor breast milk feeding for infants.

In this study, only 15% of the mothers agreed to use donated breast milk for infant feeding, and this is comparable with a report from Nigeria [21]. Like similar studies in Nigeria and South Africa, the primary reasons for their unwillingness to donate breast milk for banking or to use it for feeding infants were safety and fear of disease transmission [20, 21].

FGDs also showed that the majority of participants stated they have had received no information about human milk banking or donor breast milk feeding for infants.

Some of the participants in the FGDs mentioned that different diseases can be transmitted to infants through donor breast milk. Particularly, diseases such as cancer, acne and HIV can be transmitted. From this we understand that before the initiation of donor breast milk banking service, it is necessary to consider safety matters during the process of donation, preservation, dispensing and use of donor breast milk; and create awareness about the safety of donor breast milk among the donors and recipients.

Findings from FGDs showed that the religious issue was one of the reasons for the unwillingness to donate breast milk for banking and to accept its use for infant feeding. The study participants mentioned that it was not right to feed another mother’s breast milk for infants feeding rather it is better to feed formula or cow milk by bottle as an alternative. Some of the women also mentioned that the use of another mother’s breast milk for the infants feeding can only be possible if recipient’s baby has a blood relationship with the donor. This information may be related to the religious belief that the person who donates breast milk for a baby is considered as maternally related to the baby. Therefore, the infant would be considered as the donor’s child and marriage between the recipient’s and donor’s offspring is forbidden, as was mentioned in a paper on the introduction of donor milk banking in a Muslim country [28].

Although the majority of the participants did not support the idea of breast milk donation for banking and were not willing to use it for infant feeding, some women appreciated the advantages for mothers who could not breastfeed and the benefits for babies. The breast milk provides the best nutrition for infants especially during their first six months of life. The World Health Organization (WHO) strongly recommends that for infants who cannot receive their own mother’s breast milk, the preferred option should be donor breast milk [11].

The study showed that there are factors that can be used to increase willingness to use human breast milk banking. It was found that awareness about donor breast milk banking and wet-nurses were significantly associated with acceptance of donor milk banking. It may be that as mothers receive information related to human breast milk banking and use of someone else’s breast milk for infant feeding, their acceptance of donor breast milk increases. The study conducted by Meneses et al. in Brazil in 2013 also showed that information about breast milk expression was significantly associated with human milk donation for primary health care (Adjusted Prevalence Ratio 3.6; 95% CI 1.48, 8.97) [29].

Other factors which showed significant association with the acceptance of donor breast milk banking were the mothers’ NICU and antenatal care visits and maternal counseling about breastfeeding. This finding contradicts a study conducted in Brazil which showed a 90% reduction of breast milk donation among the participants who admitted their newborn infant to neonatal intensive care unit [29]. This might be related to maternal exposure to actual breast milk donation because in the case of the previous study those mothers might face negative effects of preterm birth that limit their milk donation tendency, whereas in the current study mothers were not exposed to actual milk donation rather they were only asked about their future willingness to donate.

The limitations of this study are that the views of health professionals and policy makers were not collected, as they are the immediate stakeholders for the implementation of human milk banking service.

## Conclusions

In conclusion, this study showed that the acceptance of breast milk donation for banking and its use for feeding infants was very low, due to lack of information and misconceptions about the safety of breast milk, along with religious reservations. Therefore, before the initiation of donor milk banking services, a program should be designed to create awareness about donor milk banking among donors and recipients.

## Abbreviations

ANC: Antenatal Care
EDHS: Ethiopian Demographic and Health Survey
FGDs: Focused Group discussions
HBMB: Human Breast Milk Banking
HIV: Human Immunodeficiency Virus
IHRERC: Institutional Health Reach Ethical Review Committee
MCH: Maternal and Child Health care
MMR: Maternal Mortality Rate
NICU: Neonatal Intensive Care Units
NMR: Neonatal Mortality Rate
PDBM: Pasteurized Human Milk Banking.
SD: Standard Deviation
SRS: Simple Random Sampling
SSA: Sub-Saharan Africa
U5MR: Under-five Mortality Rate
UNICEF: United Nations International Children’s Emergency Fund
WHO: World Health Organization

## References

[1] Arnold LDW (2006). Global health policies that support the use of banked donor human milk: a human rights issue. Int Breastfeed J.

[2] Haiden N, Ziegler EE (2016). Human Milk Banking. Ann Nutr Metab.

[3] American Academy of Pediatrics (2012). Breastfeeding and the use of human milk. Pediatrics.

[4] Henderson G, Anthony MY, McGuire W (2001). Formula milk versus preterm human milk for feeding preterm or low birth weight infants. Cochrane Database Syst Rev.

[5] Quigley M, McGuire W (2014). Formula versus donor breast milk for feeding preterm or low birth weight infants. Cochrane Database Syst Rev.

[6] Quigley MA, Henderson G, Anthony MY, McGuire W (2007). Formula milk versus donor breast milk for feeding preterm or low birth weight infants. Cochrane Database Syst Rev.

[7] Donovan S (2006). Role of human milk components in gastrointestinal development: current knowledge and future needs. JPediatr Nutr Gastrointest Tract Dev Funct.

[8] Patel AL, Johnson TJ, Engstrom JL, Fogg LF, Jegier BJ, Bigger HR (2013). Impact of early human milk on sepsis and health-care costs in very low birth weight infants. J Perinatol.

[9] Rønnestad A, Abrahamsen TG, Medbø S, Reigstad H, Lossius K, Kaaresen PI (2005). Late-onset septicemia in a Norwegian national cohort of extremely premature infants receiving very early full human milk feeding. Pediatrics.

[10] World Health Organization, UNICEF. Global strategy for infant and young child feeding. World Health Organization; 2003.

[11] World Health Organization. Guidelines on optimal feeding of low birth-weight infants in low- andmiddle-income countries. World Health Organization; 2011. 26042325

[12] Schanler RJ, Shulman RJ, Lau C (1999). Feeding Strategies for Premature Infants: Beneficial Outcomes of Feeding Fortified Human Milk Versus Preterm Formula.

[13] CSA, Central Statistical Agency (2011). Ethiopia Demographic and Health Survey, in Central Statistical Agency and ICF International C.

[14] Mekonnen Y, Tensou B, Telake DS, Degefie T, Bekele A (2013). Neonatal mortality in Ethiopia: trends and determinants. BMC Public Health.

[15] PATH. Strengthening Human Milk Banking: A Global Implementation Framework. Version 1.1. Seattle: Bill & Melinda Gates Foundation Grand Challenges initiative, PATH; 2013.

[16] World Data Bank: World development indicators. The World Bank Group; 2013. http://www.worldbank.org/en/country/ethiopia.

[17] Arslanoglu S, Moro GE, Bellù R, Turoli D, De Nisi G, Tonetto P (2013). Presence of human milk bank is associated with elevated rate of exclusive breastfeeding in VLBW infants. J Perinat Med.

[18] Bhutta ZA, Black RE (2013). Global maternal, newborn, and child health so near and yet so far. N Engl J Med.

[19] Black RE, Victora CG, Walker SP, Bhutta ZA, Christian P, De Onis M, Ezzati M (2013). Maternal and child undernutrition and overweight in low-income and middle-income countries. Lancet.

[20] Coutsoudis I, Petrites A, Coutsoudis A (2011). Acceptability of donated breast milk in a resource limited south African setting. Int Breastfeed J.

[21] Okonkwo I (2015). Mothers’ perception of the use of banked human milk for feeding of the infants. Niger J Paed.

[22] Harari Regional Health Bureau Hospitals in Harari Regional State. 2015 Harar.

[23] Harari Health Bureau. Harari Region Ethiopia [Internet]. hospital by. Available from: http://www.hospitalby.com/ethiopia-hospital/harar-hospital/ Accessed 3 Oct 2015.

[24] Jijiga Health Bureau, Jijiga General Hospital. 2009.

[25] Dil-Chora Hospital. http://placesmap.net/ET/Dil-Chora-Referral-Hospital-1343/ Accessed 3 Oct 2015.

[26] Ighogboja IS, Olarewaju RS, Odumodu CU, Okuonghae HO (1995). Mothers’ attitudes towards donated breastmilk in Jos, Nigeria. J Hum Lact.

[27] Ethiopian Federal Ministry of Health, Neonatal Intensive Care Unit (NICU) training manual 2014.

[28] Al-Naqeeb NA, Azab A, Eliwa MS, Mohammed BY (2000). The introduction of breast milk donation in a Muslim country. J Hum Lact.

[29] Meneses TM, Oliveira MIC, Boccolini CS (2017). Prevalence and factors associated with breast milk donation in banks that receive human milk in primary health care units. J Pediatr.

